# Clinical outcomes in kidney transplant recipients receiving tixagevimab/cilgavimab for outpatient treatment of COVID-19: a single-center retrospective study

**DOI:** 10.3389/frtra.2025.1579226

**Published:** 2025-06-10

**Authors:** Bilgin Osmanodja, Friederike Bachmann, Mira Choi, Wiebke Duettmann, Georgios Eleftheriadis, Fabian Halleck, Marcel G. Naik, Eva Schrezenmeier, Bianca Zukunft, Klemens Budde

**Affiliations:** Department of Nephrology and Medical Intensive Care, Charité – Universitätsmedizin Berlin, Corporate Member of Freie Universität Berlin and Humboldt-Universität zu Berlin, Berlin, Germany

**Keywords:** COVID-19, COVID-19 drug treatment, kidney transplantation, immunocompromised host, observational study

## Abstract

Kidney transplant recipients (KTR) show higher morbidity and mortality from COVID-19 than the general population and have an impaired response to vaccination. Outpatient treatment with tixagevimab/cilgavimab prevented clinical deterioration in unvaccinated patients with COVID-19 during periods of Alpha and Delta dominance. Data on the clinical outcomes in KTR receiving tixagevimab/cilgavimab for outpatient treatment during Omicron dominance are scarce. We retrospectively analyzed the clinical outcomes in a single-center cohort of 102 KTR who received tixagevimab/cilgavimab for outpatient treatment of SARS-CoV-2 infection within 7 days after symptom onset between June 29, 2022, and April 4, 2023 and compared them to a historical cohort of 219 KTR, who were infected during the Omicron period, but before tixagevimab/cilgavimab treatment was employed at our institution (January 15 until June 28, 2022). The hospitalization rate and need for ICU treatment was lower in the tixagevimab/cilgavimab group compared to the control group (2.9% vs. 15.5%, *p* = 0.001, and 0% vs. 5.9%, *p* = 0.012, respectively), while there was no statistically significant difference in COVID-19 mortality between both groups (0% vs. 2.3%, *p* = 0.124). These real-world data further support that outpatient treatment with monoclonal antibodies such as tixagevimab/cilgavimab can prevent clinical deterioration in kidney transplant recipients during a period of Omicron dominance. Novel therapeutics are needed for variants for which tixagevimab/cilgavimab shows no neutralization.

## Introduction

Immunocompromised patients including kidney transplant recipients (KTR) are at increased risk for Coronavirus disease 2019 (COVID-19) related hospitalization, intensive care unit (ICU) treatment, mechanical ventilation (MV), and in-hospital death (1–5). Consequently, they require prolonged hospitalization, which translates to increased healthcare costs (1). Despite the overall improvement in morbidity and mortality over time due to vaccination and other treatments, COVID-19 poses a continuous risk to KTR due to immunoevasion and impaired vaccine response from immunosuppressive therapy (6–8). It is therefore recommended by German national guidelines to perform early antiviral therapy in all KTR infected with SARS-CoV-2 within 5–7 days (9). As of November 2024, only two antiviral agents, nirmatrelvir/ritonavir and remdesivir are available for early antiviral treatment. However, due to interactions of ritonavir with tacrolimus and ciclosporin, practically all KTR with COVID-19 need to be admitted to the hospital to receive a 3-day course of remdesivir according to the guideline. One exception are patients treated with belatacept, which does not show interactions with ritonavir and for whom outpatient treatment can be performed. However, the majority of patients in most transplant cohorts are on calcineurin inhibitor (CNI)-based immunosuppressive therapy and therefore require treatment with remdesivir. Until April 2023, tixagevimab/cilgavimab was a convenient outpatient treatment that could be administered to KTR at risk for severe COVID-19 as an alternative to remdesivir. It was one of different therapeutic anti-SARS-CoV-2-(S)-Ag monoclonal antibodies (mABs) that were used for preexposure prophylaxis and treatment of COVID-19. The other therapeutic anti-SARS-CoV-2 mABs, casirivimab/imdevimab and sotrovimab, already showed strongly reduced *in vitro* neutralization activity for Omicron subvariants BA.1 and BA.2, respectively (10, 11). Accordingly, their use was limited to few cases after these *in vitro* data became available at most institutions. In contrast, tixagevimab/cilgavimab showed some neutralization activity against BA.1 and BA.2 subvariants and was therefore used until April 2023 (11).

Treatment with tixagevimab/cilgavimab has been shown to prevent clinical deterioration in unvaccinated patients with SARS-CoV-2 infection during periods of Alpha (B.1.1.7) and Delta (B.1.617.2) dominance (12). Data on clinical outcomes in KTR receiving tixagevimab/cilgavimab for outpatient treatment during a period of Omicron dominance are scarce (13).

Benotmane et al. described the use of tixagevimab/cilgavimab in 26 high-risk patients [defined as (1) at least one comorbid condition such as age >60 years, diabetes, obesity, cardiovascular disease, or (2) unvaccinated, or (3) weak vaccine-induced humoral response] and compared it with a control group of 35 high-risk patients who did not receive tixagevimab/cilgavimab for different reasons (e.g., late diagnosis, treatment in another hospital, unavailable tixagevimab/cilgavimab, patient refusal). The authors found a reduced risk of hospitalization and need for oxygen therapy in high-risk patients who received early outpatient treatment with tixagevimab/cilgavimab. No difference in ICU hospitalization risk and mortality was observed, probably due to the small sample size. Importantly, low-risk patients showed a very low risk of hospitalization, irrespective of the treatment strategy (tixagevimab/cilgavimab or no specific treatment). Due to the retrospective design, small sample size, and lack of control for confounding factors, conclusions about the effectiveness still must be drawn with caution (13).

Further clinical use and research on tixagevimab/cilgavimab was hampered by the emergence of immunoevasive variants such as XBB that were not neutralized by tixagevimab/cilgavimab or other monoclonal antibodies (14).

The aim of this study was to provide real-world safety and effectiveness data for tixagevimab/cilgavimab treatment in KTR with COVID-19. For this purpose, we compared clinical outcomes of KTR receiving outpatient treatment with tixagevimab/cilgavimab at our transplant center with a historical control group of KTR who did not receive tixagevimab/cilgavimab during two distinct periods of Omicron dominance.

## Materials and methods

We retrospectively analyzed clinical outcomes in a single-center cohort of 102 KTR who received tixagevimab/cilgavimab for outpatient treatment of SARS-CoV-2 infection within 7 days after symptom onset between June 29, 2022 and April 4, 2023. Patients who received tixagevimab/cilgavimab more than 7 days after symptom onset, who received tixagevimab/cilgavimab after hospitalization, or who received other anti-viral treatment, were excluded from the analysis. As a control group, we selected kidney transplant recipients with SARS-CoV-2 infection during a period of Omicron dominance who did not receive treatment with tixagevimab/cilgavimab and received at least one vaccine dose before SARS-CoV-2 infection. We selected a historical cohort before treatment with tixagevimab/cilgavimab was employed at our institution (January 10, 2022 until June 28, 2022) to reduce the risk of selection bias. We compared baseline characteristics, and main outcomes such as hospitalization, ICU treatment and mortality between both groups.

Selection criteria for treatment with tixagevimab/cilgavimab were as follows: weak serological response to vaccination, assumed at <264 BAU/ml of Anti-SARS-CoV2 IgG; immunosuppression with belatacept; intense immunosuppressive regime (e.g., additional rituximab induction); higher age and other factors of decreased immunocompetence such as previous anti-rejection treatment.

Before approval of tixagevimab/cilgavimab for the treatment of COVID-19 on September 20, 2022, we administered a dose of 300 mg as approved for preexposure prophylaxis. After September 20, 2022, both the approved dose for treatment of 600 mg and a dose of 300 mg were administered. All patients were treated according to clinical standard and received follow-up in regular intervals at our transplant center, and at least annual follow-up data were obtained for all patients.

### Database

For data collection, we used our proprietary electronic health record (EHR) TBase for KTR, where the dose and timing of tixagevimab/cilgavimab treatment were recorded (15). Records of KTR infected with SARS-CoV-2 were identified using Microsoft Access 2016 (Version 16.0.5317.1000) by applying the following criteria (separately and in combination): COVID-19 diagnosis, positive SARS-CoV-2 RNA PCR, positive anti-SARS-CoV-2-N-protein antibodies, positive anti-SARS-CoV-2 rapid test, or patient-reported history of symptomatic COVID-19. We used the day of symptom onset or the day of first positive anti-SARS-CoV-2 rapid test or anti-SARS-CoV-2 RNA PCR to determine the beginning of infection and the date of the first negative anti-SARS-CoV-2 rapid test or the first asymptomatic day to determine the end of infection. We further assessed the number and date of previous vaccinations for SARS-CoV-2. Over the course of the pandemic the following vaccines were administered at our institution: BNT162b2 (Comirnaty, BioNTech/Pfizer, Mainz, Germany), mRNA-1273 (Spikevax, Moderna Biotech, Madrid, Spain), ChAdOx1-S (AZD1222, AstraZeneca, Södertälje, Sweden), or Ad26.COV2.S (Johnson & Johnson, Janssen, Beerse, Belgium). We retrieved key clinical data, including donor demographics and available medical data from the donor reports, demographical and medical data from the recipients including laboratory values from the recipients (creatinine in mg/dl, urine-albumin-creatinine ratio in mg/g, C-reactive protein levels in mg/l, and anti-SARS-CoV-2 antibodies as described below), radiographic findings and medical reports from the EHR. Baseline estimated glomerular filtration rate (eGFR) was calculated from the most recent creatinine values before the index SARS-CoV-2 infection using the CKD-EPI-2021 formula (16). To determine whether infiltrates were solely due to COVID-19 or bacterial superinfection was present, the radiological report, laboratory values and the medical notes of the treating physicians were reviewed by a study physician (B.O.).

For quantification of serological response, the electrochemiluminescence immunoassay (ECLIA, Elecsys, Anti-SARS-CoV-2, Roche Diagnostics GmbH, Mannheim, Germany) was used. As suggested by Caillard et al., <264 BAU/ml were considered as weak serological response to vaccination (17).

### Analytical approach

The primary endpoint was COVID-19 related hospitalization. The secondary endpoints were COVID-19-related death and ICU treatment. We compared the main outcomes using a chi-square test. All statistical analyses were performed using R, version 4.1.2.

### Ethical considerations

The ethics committee of Charité–Universitätsmedizin Berlin approved this study (EA1/256/22). Patients provided informed consent for off-label treatment if applicable, but no informed consent was necessary for this retrospective analysis according to the ethics committee and German legislation. All clinical activities being reported are consistent with the principles of the Declaration of Istanbul as outlined in the “Declaration of Istanbul on Organ Trafficking and Transplant Tourism” and this study was conducted in accordance with the Declaration of Helsinki.

## Results

### Patient characteristics

From June 29, 2022 to April 4, 2023, 102 KTR received outpatient treatment with tixagevimab/cilgavimab due to SARS-CoV-2 infection and an increased risk of severe disease. 89 patients (87%) received a reduced dose of 300 mg tixagevimab/cilgavimab, while 13 patients (13%) received the approved dose of 600 mg tixagevimab/cilgavimab because of varying institutional recommendations throughout the observation period. No serious adverse events were observed after application.

99 of 102 patients received a complete SARS-CoV-2 vaccination regimen with a median of four vaccination doses (IQR 3–4) before treatment with tixagevimab/cilgavimab. 9 of 102 patients had been previously infected with COVID-19, 3 of which (33%) were previously hospitalized. 3 of 102 patients have also received preexposure prophylaxis with tixagevimab/cilgavimab before infection, all of whom were vaccinated. For 95 patients for whom anti-SARS-CoV-2 IgG was available, the median antibody levels were 564 BAU/ml (IQR 71.1–1,708.5), and 33/95 (35%) showed a weak response to vaccination (<264 BAU/ml of anti-SARS-CoV2 IgG) before therapy initiation. In the control group, patients received a median of 3 (IQR 3–4) vaccination doses before infection (mean 3.31 vs. mean in the tixagevimab group 3.78). Median patient age was lower in the control group than in the tixagevimab/cilgavimab group (54.5 vs. 57.8 years), while median time since transplantation was higher in the control group than in the tixagevimab/cilgavimab group (9.4 vs. 6.8 years). Kidney function parameters were comparable between both groups (median creatinine 1.46 vs. 1.46 mg/dl, median urine albumin 20.5 vs. 29 mg/g). The baseline characteristics of the study cohort are presented in Table 1.

**Table 1:** Baseline characteristics of kidney transplant recipients infected with SARS-CoV-2 during the omicron era grouped by treatment with tixagevimab/cilgavimab.

Characteristics	Tixagevimab/cilgavimab	Control
	*n* = 102	*n* = 219
Demographics		
Age	57.83 (18.76)	54.48 (20.00)
Sex female/male	40 (39%)/62 (61%)	79 (36%)/140 (64%)
BMI kg/m^2^	24.23 (5.13)	25.10 (6.01)
Transplantation		
Time since transplantation in years	6.75 (10.97)	9.35 (11.55)
Creatinine mg/dl	1.46 (0.64)	1.46 (0.69)
Albumin-creatinine ratio mg/g	20.50 (62.50)	29.00 (120.00)
Kidney transplant number		
1/2/3/4	93 (91%)/7 (7%)/2 (2%)/0	205 (94%)/12 (5%)/1 (0.5%)/1 (0.5%)
Kidney donation type		
Deceased donor	61 (60%)	98 (45%)
Living donor	40 (39%)	99 (45%)
Unknown	1 (1%)	22 (10%)
Primary disease		
Other	22 (21.6%)	65 (29.7%)
Glomerulonephritis	19 (18.6%)	27 (12.3%)
IgA Nephropathy	13 (12.7%)	20 (9.1%)
Polycystic kidney disease	13 (12.7%)	26 (11.9%)
Diabetic nephropathy	4 (3.9%)	10 (4.6%)
Unknown	31 (30.4%)	71 (32.4%)
Number of vaccinations before infection/pre-exposure prophylaxis		
0	3 (2.9%)	0 (0%)
1	1 (1%)	6 (2.7%)
2	9 (9%)	37 (16.9%)
3	26 (25%)	97 (44.3%)
4	38 (37%)	53 (24.2%)
5	17 (17%)	18 (8.2%)
6	5 (5%)	6 (2.7%)
7	3 (3%)	2 (0.9%)

Median (IQR) or *n* (%).

### Outcomes

From 102 patients with SARS-CoV-2 infection who received outpatient treatment with tixagevimab/cilgavimab, 3 patients (2.9%) were hospitalized, none of which required ICU treatment, and no patient died. Median disease duration was 21 days (IQR 15–29) and was assessed in 55/102 patients (54%). From the 3 patients that were hospitalized, 2 patients showed clinical and radiological signs of COVID-19-related pneumonia, one of which also showed signs of bacterial superinfection with strongly elevated levels of C-reactive protein. The patient with bacterial superinfection was treated with intravenous antibiotics with complete resolution of pneumonia and did not receive additional COVID-19-specific therapy. The other patient with COVID-19-related pneumonia was treated with a 3-day course of remdesivir additionally to the previous treatment with tixagevimab/cilgavimab. The third patient was hospitalized due to frailty and inability to perform the activities of daily living due to COVID-19 infection. In this patient, no additional COVID-19 specific therapy was performed, since no signs of COVID-19-related organ disfunction were present. All patients were discharged from the hospital without relevant sequelae. From the remaining 99 patients that were not hospitalized, 5 patients underwent chest imaging due to clinical indication at the time of infection (1 CT, 4 x-ray), of which one showed clinical and radiographical signs of COVID-19-related pneumonia in x-ray that did not require hospitalization or additional medical treatment. From 219 patients in the control group, 34 patients (15.5%) were hospitalized, 24 due to COVID-related pneumonia, 10 due to inability to perform ADL. In the control group, 13/219 patients (5.9%) required ICU treatment, and 5 patients (2.3%) died due to COVID-19.

**Figure 1:**
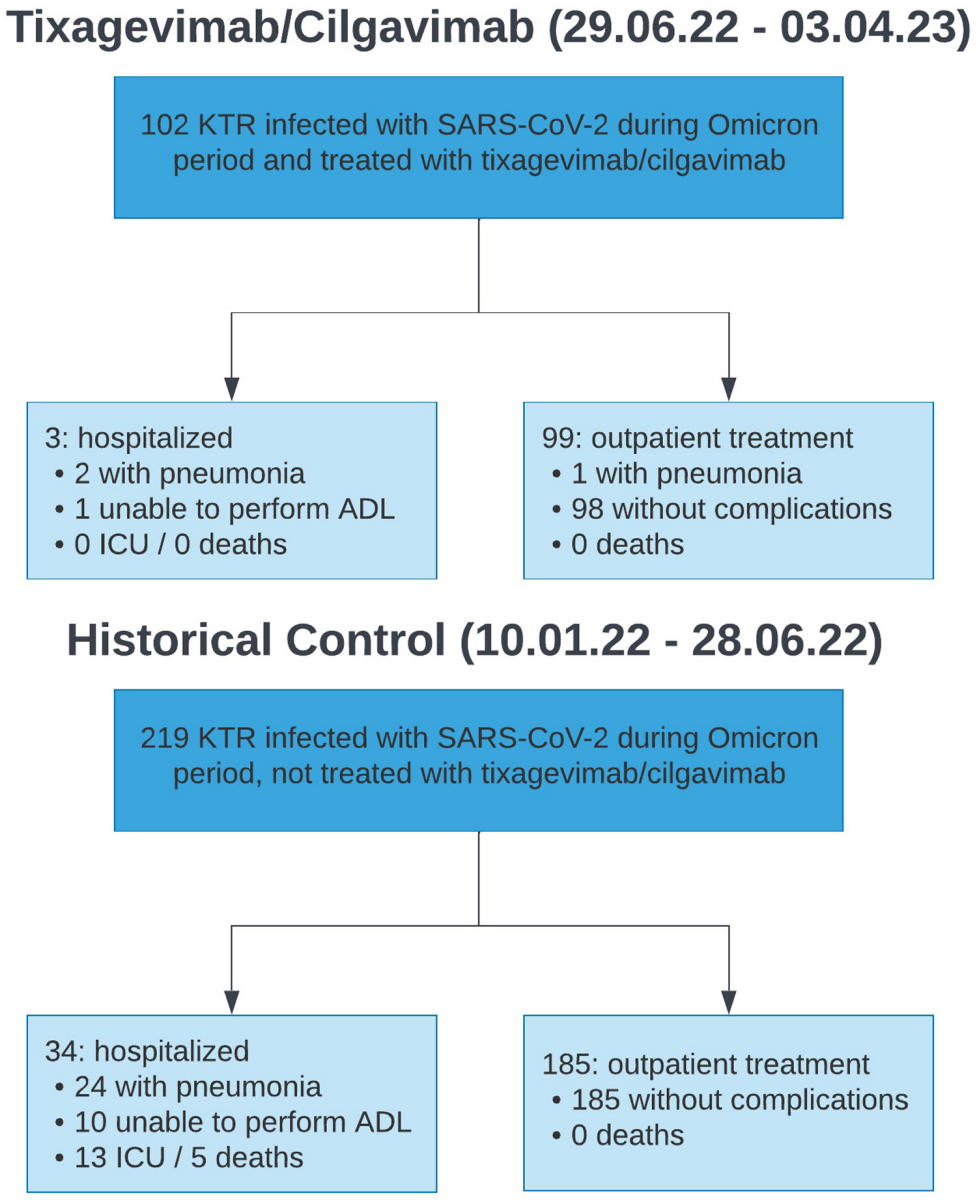
Patient flow chart showing main outcomes grouped by intervention (tixagevimab/cilgavimab) and control (historical cohort before treatment with tixagevimab/cilgavimab was performed). KTR, kidney transplant recipients; COVID-19, Coronavirus-disease 2019; ADL, activities of daily living; ICU, intensive care unit treatment.

The hospitalization rate and need for ICU treatment was lower in the tixagevimab/cilgavimab group compared to the control group (2.9% vs. 15.5%, *p* = 0.001, and 0% vs. 5.9%, *p* = 0.012, respectively), while there was no statistically significant difference in COVID-19 mortality between both groups (0% vs. 2.3%, *p* = 0.124). The main results are summarized in Figure 1 and Table 2.

**Table 2:** COVID-19 outcomes in kidney transplant recipients infected with SARS-CoV-2 during the omicron era grouped by treatment with tixagevimab/cilgavimab.

COVID-19 outcomes	Tixagevimab/cilgavimab	Control group (vaccinated)	*p* value
	*n* = 102	*n* = 219	(Chi-square test)
Hospitalisation	3 (2.9%)	34 (15.5%)	**0** **.** **001**
ICU treatment	0 (0%)	13 (5.9%)	**0** **.** **012**
COVID-19 related death	0 (0%)	5 (2.3%)	0.124

*n* (%). ICU, intensive care unit.

Bold values indicating significant differences with *p* < 0.05.

### Antibody titers after treatment

Anti-SARS-CoV-2 IgG titers were available for 28 patients at 4–6 months after treatment with tixagevimab/cilgavimab. The majority of 22/28 of these patients (79%) had titers >2,500 BAU/ml, above which no further quantification was performed at our laboratory. Only one patient (4%) had a titer <264 BAU/ml during a 6 months follow-up. For a subgroup of 18 patients, anti-SARS-CoV-2 IgG titers were also available at later time points (mean 9.1 months after treatment, standard deviation 2.9). The majority of 15/18 of these patients (83%) had titers >2,500 BAU/ml, while one patient (6%) had a titer <264 BAU/ml.

## Discussion

In this article, we show that outpatient treatment with tixagevimab/cilgavimab in KTR infected with SARS-CoV-2 during the Omicron period was associated with a lower hospitalization rate and lower rate of ICU treatment than in a historical control group consisting of KTR infected with Omicron before tixagevimab/cilgavimab was used for outpatient treatment at our instititution.

The outcomes we observed in the tixagevimab/cilgavimab group are comparable to the excellent outcomes described by Benotmane et al., who observed a hospitalization rate of 3.8% and mortality rate of 0% in 26 high-risk patients treated with tixagevimab/cilgavimab for COVID-19. They also compared these outcomes to a control group of 35 patients who were not treated with tixagevimab/cilgavimab, among whom the hospitalization rate was 34%, the ICU admission rate was 14.3% and mortality was 8.6% (13). Despite the fact that the cases in our control group were less severe than those reported by Benotmane et al. (hospitalization: 15.5% vs. 34%, ICU: 5.9% vs. 14.3%, mortality 2.3% vs. 8.6%), we still found a significant difference between patients treated with tixagevimab/cilgavimab and the control group with respect to hospitalization and ICU treatment. In contrast to Benotmane et al., we used a historical control group (within the Omicron period) to reduce the risk for selection bias. Interestingly, the outcomes in the tixagevimab/cilgavimab group are roughly comparable to the case fatality rates reported for the general population in Germany during a Delta/Omicron period of 0.39% (18).

Regarding patient selection, Benotmane et al. suggested that among low-risk patients who were vaccinated, had a SARS-CoV-2 antibody titer >264 BAU/ml, were younger than 60 years, and did not have certain comorbidities, due to low overall risk for hospitalization, therapy with tixagevimab/cilgavimab was not necessary and was therefore seldom performed (13). In contrast, we treated patients more liberally with tixagevimab/cilgavimab at our institution for two reasons. On the one hand, data from Benotmane et al. became available only after all patients in this study were treated. On the other hand, there was increasing evidence that a relevant proportion of patients with antibody levels above the cut-off of 264 BAU/ml did not exhibit relevant neutralization of Omicron subvariants (8). In neutralization assays using sera of vaccinated and infected KTR, Moal et al. demonstrated that only 46% of KTR neutralized Delta and all tested Omicron subvariants (BA.1, BA.2, BA.4, BA.5), while the remaining 54% showed no neutralization for at least one variant, and no neutralization was observed in 18% of KTR, regardless of the variant (8). However, this stricter selection may be one reason for the higher case severity by Benotmane et al. discussed above.

While data for outpatient treatment of SARS-CoV-2 infection with tixagevimab/cilgavimab are scarce, several articles support the use of tixagevimab/cilgavimab preexposure prophylaxis for the prevention of symptomatic COVID-19 in KTR (19–22). Most recently, Reindl-Schwaighofer et al. demonstrated that tixagevimab/cilgavimab preexposure prophylaxis resulted in a reduced COVID-19 incidence and lower rate of severe COVID-19 during periods of Omicron BA.2 dominance, but not during BA.4/5 dominance. They also provided pharmacokinetic data on tixagevimab/cilgavimab showing comparable results to the general population (22).

Given the emergence of novel variants starting with XBB in early 2023, which have diminished virus neutralization by tixagevimab/cilgavimab, and the persistent risk of COVID-19 in immunocompromised patients such as KTR, there is an urgent and unmet need to develop novel therapeutics to address this challenge. In the SUPERNOVA phase I/III trial of sipavibart for the prevention of symptomatic COVID-19 immunocompromised patients, safety and efficacy have been shown recently (23). Sipavibart is an investigational long-acting monoclonal antibody (LAAB) against SARS-CoV-2 that is designed to provide broad neutralization across various subvariants.

Since no approved therapeutic monoclonal antibody for early outpatient treatment of COVID-19 is available as of November 2024, patients at high risk for severe COVID-19 should be treated with antiviral agents according to German national guidelines (9). These include patients after solid organ transplantation, treatment with anti-B cell antibodies (e.g., rituximab) as long as no reconstitution of B cell capacity has occurred, chimeric antigen receptor (CAR) T cell therapy, and other severe immunosuppression (ongoing chemotherapy, severe immune deficiencies, as well as autologous or allogeneic stem cell transplantation). Due to the interactions of nirmatrelvir/ritonavir with CNI and the de facto inability to use this medication for outpatient treatment in the majority of KTR, most patients need to be admitted to the hospital to receive a 3-day course of remdesivir in adherence with German guidelines so far. This underscores the importance of an efficacious and convenient early outpatient treatment for high-risk patients with COVID-19.

## Limitations

Due to the diminished neutralization by tixagevimab/cilgavimab seen in novel SARS-CoV-2 variants, these data are historical and have no current clinical implication. The differences in hospitalization and ICU treatment rates between tixagevimab/cilgavimab need to be interpreted with caution, since there are several limitations including the single-center design, group imbalance, inability to adjust for treatment in the control group, and differences in baseline characteristics (e.g., age, time since transplantation, vaccination frequency). We decided to include only patients with at least one vaccination in the control group, since mortality and morbidity in unvaccinated KTR are especially high, which could strongly bias the results. Still, patients in the control group had less vaccinations than patients in the tixagevimab/cilgavimab group, which is a risk for bias. While the decision to use a historical cohort reduces the risk for selection bias, it poses the risk for bias due to differences in management and treatment success in the different periods. Due to the observational design, conclusions about efficacy of tixagevimab/cilgavimab treatment cannot be drawn. Another limitation are the varying dosing regimens throughout the observation period.

## Conclusion

This article provides additional real-world data that support the safety and effectiveness of outpatient treatment with tixagevimab/cilgavimab in kidney transplant recipients during a period of Omicron dominance. Efficacious and safe therapeutic agents for early outpatient treatment of patients at high-risk for severe COVID-19 are still needed. Currently, repeated vaccination against SARS-CoV-2 in kidney transplant patients and early antiviral treatment is recommended by German national guidelines.

## Data Availability

The original contributions presented in the study are included in the article/Supplementary Material, further inquiries can be directed to the corresponding author.
